# Concomitant targeting of programmed death-1 (PD-1) and CD137 improves the efficacy of radiotherapy in a mouse model of human BRAFV600-mutant melanoma

**DOI:** 10.1007/s00262-016-1843-4

**Published:** 2016-05-09

**Authors:** Paula Kroon, Jules Gadiot, Marlies Peeters, Alessia Gasparini, Marcel A. Deken, Hideo Yagita, Marcel Verheij, Jannie Borst, Christian U. Blank, Inge Verbrugge

**Affiliations:** 1 Divisions of Immunology, The Netherlands Cancer Institute, Plesmanlaan 121, 1066 CX Amsterdam, The Netherlands; 2 Divisions of Radiotherapy, The Netherlands Cancer Institute, Amsterdam, The Netherlands; 3 Divisions of Medical Oncology, The Netherlands Cancer Institute, Amsterdam, The Netherlands; 4 Department of Immunology, Juntendo University School of Medicine, Tokyo, Japan

**Keywords:** PD-1, CD137, Radiotherapy, Stereotactic, Melanoma, BRAFV600

## Abstract

T cell checkpoint blockade with antibodies targeting programmed cell death (ligand)-1 (PD-1/PD-L1) and/or cytotoxic T lymphocyte-antigen 4 (CTLA-4) has improved therapy outcome in melanoma patients. However, a considerable proportion of patients does not benefit *even* from combined α-CTLA-4 and α-PD-1 therapy. We therefore examined to which extent T cell (co)stimulation and/or stereotactic body radiation therapy (SBRT) could further enhance the therapeutic efficacy of T cell checkpoint blockade in a genetically engineered mouse melanoma model that is driven by PTEN-deficiency, and BRAFV600 mutation, as in human, but lacks the sporadic UV-induced mutations. Tumor-bearing mice were treated with different combinations of immunomodulatory antibodies (α-CTLA-4, α-PD-1, α-CD137) or interleukin-2 (IL-2) alone or in combination with SBRT. None of our immunotherapeutic approaches (alone or in combination) had any anti-tumor efficacy, while SBRT alone delayed melanoma outgrowth. However, α-CD137 combined with α-PD-1 antibodies significantly enhanced the anti-tumor effect of SBRT, while the anti-tumor effect of SBRT was not enhanced by interleukin-2, or the combination of α-CTLA-4 and α-PD-1. We conclude that α-CD137 and α-PD-1 antibodies were most effective in enhancing SBRT-induced tumor growth delay in this mouse melanoma model, outperforming the ability of IL-2, or the combination of α-CTLA-4 and α-PD-1 to synergize with SBRT. Given the high mutational load and increased immunogenicity of human melanoma with the same genotype, our findings encourage testing α-CD137 and α-PD-1 alone or in combination with SBRT clinically, particularly in patients refractory to α-CTLA-4 and/or α-PD-1 therapy.

**Electronic supplementary material:**

The online version of this article (doi:10.1007/s00262-016-1843-4) contains supplementary material, which is available to authorized users.

## Introduction

Cancer immunotherapy, aimed at stimulating tumor-reactive T cells to eliminate (disseminated) tumors, is a promising new treatment approach in various cancers, including melanoma, a cancer with a high (UV-induced) mutational load and potential immunogenicity [1]. Interleukin-2 (IL-2), an immunomodulatory cytokine that promotes T cell and natural killer (NK) cell activity has been tested extensively in the clinic in the 1980s and 1990s and induced long-term responses in a small group of melanoma patients, but also caused severe toxicities [2, 3]. More promising data were generated in randomized trials using T cell response modulation by monoclonal antibodies (mAbs) targeting the coinhibitory receptors CTLA-4 (ipilimumab) and PD-1 (nivolumab, pembrolizumab) alone or in combination [4–8]. However, still 40–50 % of late-stage melanoma patients does not benefit long term from these monotreatments [9, 10]. Recent studies indicate that pembrolizumab or nivolumab are more effective than ipilimumab in advanced melanoma [5, 8], yet ipilimumab could further improve anti-tumor immune responses induced by nivolumab [8], indicating α-PD-1 and α-CTLA-4 antibodies have (partially) distinct mechanisms of action and we are currently awaiting long-term benefits of these (combined) treatment modalities.

A possible reason for failure to respond to checkpoint inhibitors is the insufficient presence, recruitment and/or functionality of T cells into the tumor microenvironment [11]. Therefore, a strategy to induce effective anti-tumor immunity should combine induction of tumor-reactive T cells with interventions to overcome tumor-associated immunosuppression.

One way to enhance tumor-specific T cell responses is to use agonistic antibodies that engage T cell costimulatory receptors, including cluster of differentiation proteins (CD)137 (4-1BB), CD27 and CD134 (OX40). These antibodies have been shown to improve anti-tumor T cell responses in preclinical and early clinical studies (reviewed in [12]) and can enhance the efficacy of α-PD-1 and α-CTLA-4 therapy (e.g., [13, 14]).

Another way to potentially enhance tumor-specific T cell responses is tumor irradiation. Radiotherapy kills tumor cells locally, but can also modulate anti-tumor immune responses by converting the irradiated tumor into an in situ vaccine and by broadening the intratumoral T cell repertoire [15–17]. In addition, high-dose radiotherapy may enhance tumor cell visibility to the immune system by up-regulating MHC class I, CD80 and CD95/Fas [17–20]. It can also increase tumor infiltration by activated CD8^+^ T cells (e.g., [21]). Several preclinical studies indicate that the anti-tumor efficacy of various T cell activating immunotherapies, such as mAbs to CTLA-4, PD-1, CD137, or CD40, vaccines and adoptive T cell transfer, can be enhanced by radiotherapy (e.g., [14, 16, 17, 22–24]. However, these studies were mostly carried out in models using transplantable tumor cell lines expressing model antigens, which do not represent tumors that have spontaneously arisen in patients. The efficacy of immunotherapeutic approaches in combination with radiotherapy in *de novo* arisen tumors has not been addressed so far.

Therefore, in this study, we aimed to identify which T cell modulating antibody combinations (α-CTLA-4, α-PD-1, α-CD137) could enhance the anti-tumor effect of SBRT in an inducible mouse model of human BRAFV600-mutant and PTEN-deficient melanoma [25, 26]. This mouse model faithfully resembles human metastatic melanoma in terms of these genetic driver mutations, but not in terms of UV-induced lesions that contribute to tumor immunogenicity, resulting in low tumor immunogenicity as compared to human melanoma. We compared these immunotherapeutic combinations to the currently most promising combination in the clinic, namely SBRT with IL-2 [27]. We found that the combination of PD-1 blocking and CD137 agonism was most effective in enhancing the anti-tumor effect of SBRT, which was dependent on both CD4 and CD8 T cells. Therefore, concomitant targeting of PD-1 and CD137 in combination with SBRT may be attractive for clinical testing.

## Materials and methods

### Mice, tumor induction and growth analysis

Tumors were induced on the skin of C57Bl/6J *Tyr::CreER* ^*T2*^ *;Pten* ^*loxP/loxP*^ *;Braf* ^*CA/*+^ mice as previously described [25, 26, 28]. In these mice, the estrogen receptor (ER) ligand tamoxifen induces expression of mutant Braf and loss of Pten in melanocytes. Briefly, 2 μl of 5-mM 4-hydroxytamoxifen (4-OHT, Sigma-Aldrich, H6278) in pure DMSO (Sigma-Aldrich, 276855) was applied topically on the flank of 4- to 8-week-old mice. Tumor outgrowth was monitored twice weekly by digital photographs of the tumor with a size reference. Tumor size was subsequently analyzed in two dimensions using ImageJ software (developed by the National Institutes of Health, USA). Mice were maintained under specific pathogen-free conditions. All mouse experiments were performed in accordance with institutional and national guidelines and were approved by the Animal Experimental Committee of the Netherlands Cancer Institute.

### Therapeutic antibodies and reagents

Rat α-mouse CD137 mAb (3H3, IgG2a) [29], derived from hybridoma culture supernatant, was protein-G purified. Rat α-mouse PD-1 mAb (RMP1-14; IgG2a) [30] was purchased from BioXCell. 2A3 mAb (BioXCell) was used as an isotype Control. Mouse α-mouse CTLA-4 mAb (9D9) was from BioXCell, and IL-2 (Proleukin) was from Novartis.

### Tumor therapy

Therapy (5–10 mice per group) commenced when tumors reached ~20 mm^2^. Radiotherapy of melanomas was conducted as described using the XRAD225-Cx system (Precision X-Ray Inc., CT, USA [22]). Briefly, mice were anesthetized with isoflurane after which a cone-beam CT scan of the mice was generated. Tumors were localized on the computed tomography (CT) scan and targeted for radiotherapy with 0.1-mm accuracy using round collimators of 1.0 or 1.5 cm in diameter. A single fraction of 14 Gy (225 peak kilovoltage (kVp), filtered with 0.3 mm of copper, 3 Gy/min) was delivered. Control mice were anesthetized and underwent a cone-beam CT scan, but were not exposed to radiotherapy.

Immunomodulatory α-PD-1, α-CD137, α-CTLA-4 or Control 2A3 mAbs diluted in PBS were administered at 100 μg/mouse intraperitoneally twice weekly for 2 weeks with the first dose delivered immediately after radiotherapy. IL-2 (in PBS) was administered as a high dose (7.2 × 10^5^ IU) twice daily for three consecutive days starting 3 days following radiotherapy. This dosing schedule was chosen to mimic the dosing schedule used in clinical trials as closely as possible [27]. Mice were killed when tumors reached 100–200 mm^2^. A tumor size of 100 mm^2^ was set as designated end point.

For the determination of tumor size at different time points post-treatment were the (interpolated) tumor sizes taken of all analyzable mice in each group. In case the tumor reached >100 mm^2^ at a time point analyzed, the value was set to 100 mm^2^. The survival curves generated represent the fraction of mice bearing tumors smaller than 100 mm^2^. Censored events indicate mice that were killed before treated tumors reached 100 mm^2^.

### Phenotyping of lymphocytes resident in tumor and peripheral lymphoid tissue

Mice bearing 2–3 established melanomas were subjected to 14 Gy localized radiotherapy to one of the tumors. One week later, single-cell suspensions were prepared from non-irradiated tumors, irradiated tumors or inguinal lymph nodes, stained with the fluorochrome-conjugated mAbs (from BD Pharmingen unless otherwise specified) indicated below and analyzed by flow cytometry according to the following gating strategy: Single-cell suspensions were gated on live (DAPI negative) cells. α-CD45-PE-Cy7 (104) was used to discriminate tumor (CD45^−^) and immune (CD45^+^) cells. NK cells, CD4 and CD8 T cells were identified using α-TCRβ-PE-Cy5 (H57-597), α-CD8-PerCpCy5.5 (53.6.7; CD8^+^ T cells: TCRβ^+^CD8^+^), α-CD4-FITC (GK1.5; CD4^+^ T cells: TCRβ^+^ CD4^+^), α-NK1.1-allophycocyanin (APC)-Cy7 (PK136; NK cells: TCRβ-NK1.1^+^). On each of these immune cell populations, CD25 (PC61), CTLA-4 (UC10-4F10-11) and PD-1 (J43; eBioscience) were detected using indicated PE-conjugated antibodies and CD137 was detected using a biotinylated antibody (17B5; eBioscience) followed by APC-conjugated streptavidin. This staining strategy allowed us to examine co-expression of CD137 and any of CD25, PD-1 and CTLA-4 in any of the CD4 T cell, CD8 T cell and NK cell subsets. Biotinylated or PE-conjugated isotype Controls were included for stainings to CD137, PD-1, CTLA-4 and CD25. The frequency of positive cells was determined by subtracting the % positive fraction in isotype Control staining from the % positive fraction in the staining specific for CD25, PD-1, CTLA-4 or CD137. This number is indicated in the graphs and was used for statistical analyses. CD137 staining was analyzed in triplicate in each sample and was averaged for statistical analysis. Numbers in text represent mean ± SEM. Samples were analyzed on a BD Fortessa. Examples of gating strategies for lymph node and tumor are presented in Supplemental Figs. 1 (lymph node) and 2 (tumor).

**Fig. 1:**
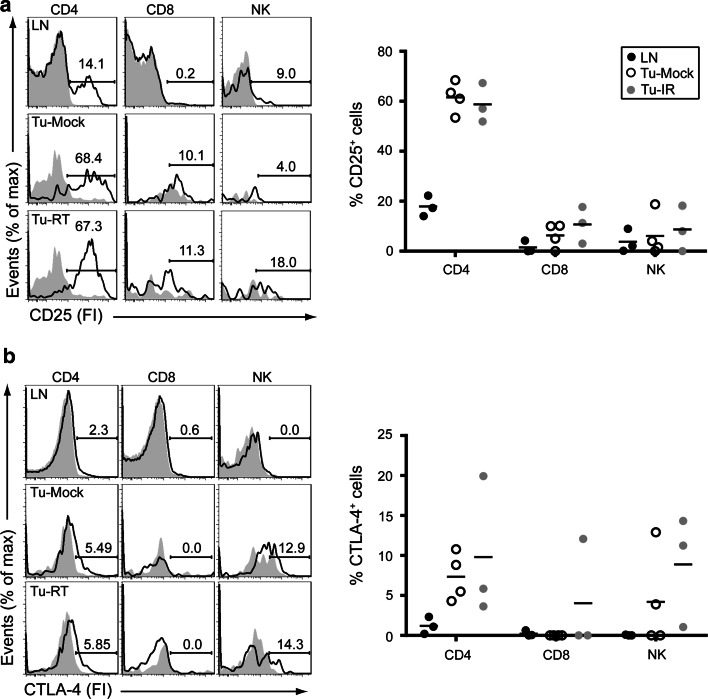
TILs express surface CD25 and CTLA-4. Mice bearing established melanomas were killed 1 week following 14 Gy SBRT. Single-cell suspensions were prepared from inguinal lymph nodes (LNs), non-irradiated tumors (Tu-Mock) and irradiated tumors (Tu-RT). Flow cytometric analysis of (**a**) CD25 and (**b**) CTLA-4 expression on gated CD4^+^ (TCRβ^+^CD4^+^), CD8^+^ (TCRβ^+^CD8^+^) T cells and NK (TCRβ^−^NK1.1^+^) cells from tumor or lymph node (LN) as indicated. *Solid* histograms represent isotype-matched Control antibodies and open histograms CD25, or CTLA-4-specific surface staining from an individual sample. *Numbers* indicate % of positive cells. *Right panels* indicate quantification of 3–4 individual mice

### Immunohistochemical analysis

For immunohistochemical analysis, tumors (three mice per group) were fixed for 24 h in ethanol (50 %), acetic acid (5 %), formalin (3.7 %), embedded in paraffin, randomly sectioned at 4 µm. Staining was performed as previously described [31]. Briefly, fixed sections were rehydrated and incubated with primary antibodies. Endogenous peroxidases were blocked with 3 % H_2_O_2_ and stained with biotin-conjugated secondary antibodies, followed by incubation with HRP-conjugated streptavidin–biotin complex (DAKO). Substrate was developed with either 3-amino-9-ethylcarbazole (AEC) or diaminobenzidine (DAB) (DAKO). Primary antibodies were α-CD3 (clone SP7, cat. RM-9107 Thermo Scientific), α-CD4 (cat. 14-9766 eBioscience), α-FoxP3 (cat. 14-5773 eBioscience).

CD8 staining was performed on optimal cutting temperature compound (OCT) embedded, cryopreserved tumor pieces using standard procedures. Briefly, tumor pieces were thawed to room temperature, rehydrated in PBS and blocked for avidin and biotin (Vector SP-2001). After sections were blocked in 5 % normal goat serum and 2.5 % BSA, sections were incubated for 1 h with primary α-CD8 antibody (clone 2.43). After washing, sections were incubated with biotinylated secondary antibodies, followed by incubation with HRP-conjugated streptavidin–biotin complex and substrate was developed with DAB.

Slides were counterstained with hematoxylin and slides scanned using the Aperio ScanScope (Leika) (20× objective). ImageJ software was used to quantify # positive cells (CD3, CD4, FoxP3) or % positive area (CD8) from 3 to 5 random fields of view (FOV) per slide.

### Statistics

Statistical differences between groups were analyzed with the Mann–Whitney *U* test using GraphPad Prism (GraphPad Software) and considered significant when *p* < 0.05.

## Results

### Tumor-infiltrating lymphocytes (TILs) express CD25, CTLA-4, PD-1 and CD137

First, we investigated whether relevant cell surface receptors were available for targeting on melanoma tumor-infiltrating lymphocytes (TILs) before and after radiotherapy. For this purpose, CD4^+^ and CD8+ T cells and natural killer (NK) cells were examined for expression of CD25 (IL-2 receptor α-chain), CTLA-4, PD-1 and CD137. Examination of CD25 was chosen because of potent combined effects of SBRT and IL-2 in the clinic [27]; CTLA-4 and PD-1 because of potent (combined) efficacy of α-CTLA4 and α-PD-1 mAbs in late-stage melanoma patients [32] and CD137 because of potent combined effects of α-CD137/α-PD-1 mAbs and SBRT in mouse breast cancer models [14, 22].

In mice bearing 2–3 melanomas, one of these tumors was subjected to 14 Gy SBRT. Pilot experiments revealed that this radiotherapy dose induced tumor growth delay of irradiated tumors without inducing complete tumor regression. Therefore, this dose provided a window to read out the combined effect of immune-modulatory agents on radiotherapy-induced tumor growth delay.

One week after radiotherapy, single-cell suspensions were prepared from irradiated and non-irradiated tumors of the same mice, as well as from their (inguinal) lymph nodes and flow cytometric detection of receptors was performed (See Supplemental Figures 1 and 2 for gating).

CD25 was expressed in non-irradiated tumors (Tu-mock) on the majority of CD4^+^ T cells (60.8 ± 2.6 %), on small populations of CD8^+^ T cells (6.0 ± 2.7 %) and NK cells (6.1 ± 5.6 %). Radiotherapy did not significantly alter this expression. In lymph nodes, CD25 was expressed on a subset of CD4^+^ T cells (17.9 ± 2.4 %) and on a small proportion of NK cells (4.8 ± 3.7 %) and was hardly found on CD8^+^ T cells (1.5 ± 1.4 %; Fig. 1a).

CTLA-4 was expressed in non-irradiated tumors on a proportion of CD4^+^ T cells (7.4 ± %1.5 %), a subset of NK cells (4.2 ± 3.1 %), but not on CD8^+^ T cells. In irradiated tumors, CTLA-4 was occasionally detected on CD8^+^ T cells (2.5 ± 4.9 %). In lymph nodes, CTLA-4 was only expressed on a small percentage (1.2 ± 0.6 %) of CD4^+^ T cells (Fig. 1b).

**Fig. 2:**
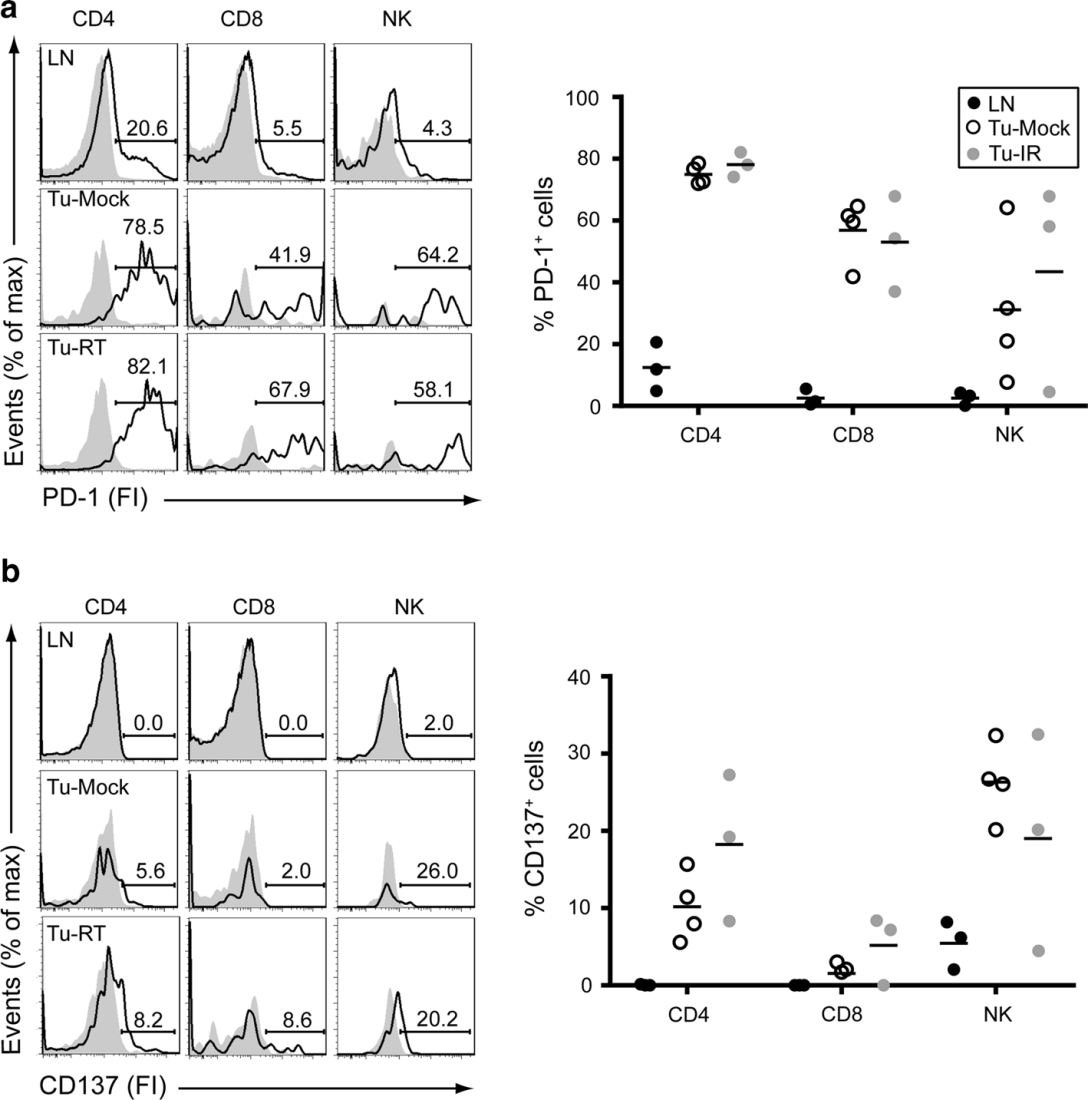
TILs express surface PD-1 and CD137. Single-cell suspensions of inguinal lymph nodes (LNs), non-irradiated tumors (Tu-Mock) and irradiated tumors (Tu-RT) as in Fig. 1 were also analyzed for expression of PD-1 and CD137. Flow cytometric analysis of (**a**) PD-1 and (**b**) CD137 expression on gated CD4^+^ (TCRβ^+^CD4^+^), CD8^+^ (TCRβ^+^CD8^+^) T cells and NK (TCRβ^−^NK1.1^+^) cells as indicated. Solid histograms represent isotype-matched Control antibodies and open histograms PD-1 or CD137-specific surface staining from an individual sample. *Numbers* indicate % of positive cells. *Right panels* indicate quantification of 3–4 individual mice

PD-1 was expressed in non-irradiated tumors on the majority of both CD4^+^ and CD8^+^ T cells (means 53.0–78.1 %) and on NK cells (25.6 ± 7.3 %). Radiotherapy did not significantly alter PD-1 expression. Expression of PD-1 in lymph nodes was found to a lesser extent as compared to TILs (CD4^+^ T cells: 12.4 ± 4.6 %, CD8^+^ T cells: 2.5 ± 1.5 %, NK cells: 2.6 ± 1.3 %, Fig. 2a).

CD137 was detected in non-irradiated tumors on CD4^+^ T cells (10.17 ± 2.2 %), a small fraction of CD8^+^ T cells (1.5 ± 0.8 %) and a sizable fraction of NK cells (26.5 ± 2.5 %). Radiotherapy slightly increased the frequency of CD137-expressing CD4^+^ and CD8^+^ T cells, but this did not reach statistical significance. In lymph nodes, CD137 expression was detected on a fraction of NK cells (5.9 ± 1.4 %), but expression on CD4^+^ and CD8^+^ T cells was negligible (Fig. 2b).

Similar data were obtained when TILs were analyzed 2 days after radiotherapy (Supplemental Figure 3). Due to the small number of T cells recovered from these tumors, the variability within the groups is relatively large, leaving us unable to draw strong conclusions. However, we again observed no significant differences between TILs in irradiated versus non-irradiated tumors.

Foxp3, the hallmark protein of regulatory T cells (Tregs) was detected in 30–75 % of the intratumoral CD4^+^ T cells. Their frequency was not significantly altered by radiotherapy (Supplemental Figure 4a). In addition, radiotherapy did not alter the frequency of CD4^+^ T cells, CD8^+^ T cells and NK cells (Supplemental Figure 4b). CTLA-4, CD25 and CD137 were expressed on a higher frequency of regulatory (Foxp3+) CD4 TILs cells than on ‘non-regulatory’ (Foxp3−) CD4 TILs cells, whereas PD-1 expression was similar between the two subsets. This was not significantly affected by radiotherapy (Supplemental Figure 5).

The presence of CD25, CTLA-4, PD-1 and/or CD137 on T cells and NK cells indicates that IL-2 or antibodies targeting these receptors may have an immune modulatory and potentially therapeutic effect. We therefore tested the effectiveness of IL-2 or mAbs targeting CTLA-4, PD-1 and CD137 alone and in combination with SBRT.

### Concomitant targeting of CD137 and PD-1 enhances the therapeutic efficacy of SBRT

Mice bearing established (>20 mm^2^) tumors were divided into the following treatment groups that consisted of immunotherapy alone or in combination with 14 Gray (Gy) SBRT: (1) Control Ig (Ctr), (2) IL-2, (3) α-PD-1 and α-CTLA-4, (4) α-PD-1, (5) α-CD137, (6) α-PD-1 and α-CD137, (7) SBRT, (8) SBRT + IL-2, (9) SBRT + α-PD-1 and α-CTLA-4, (10) SBRT + α-PD-1 and α-CD137, (11) SBRT + α-PD-1 and (12) SBRT + α-CD137.

**Fig. 3:**
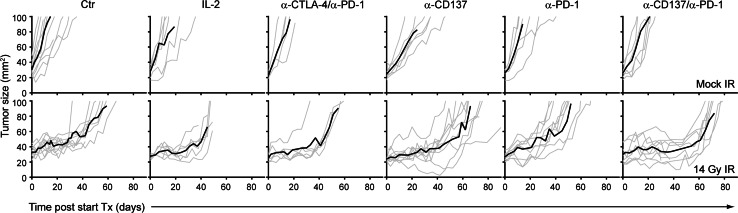
α-CD137 and α-PD-1 immunotherapy enhances the therapeutic efficacy of radiotherapy in melanoma. Tumor growth curves of mice (5–11 per group) bearing established (>20 mm^2^) melanomas that were treated with 14 Gy radiotherapy (bottom panels) or mock-irradiated (top panels) in combination with IL-2, α-CTLA-4 and α-PD-1, α-CD137 and/or α-PD-1 or isotype-matched Control antibody (Ctr) as indicated. Individual tumor growth curves (*gray lines*) and mean tumor growth (*black line*) are shown. Results shown are accumulated data from five separate experiments

**Fig. 4:**
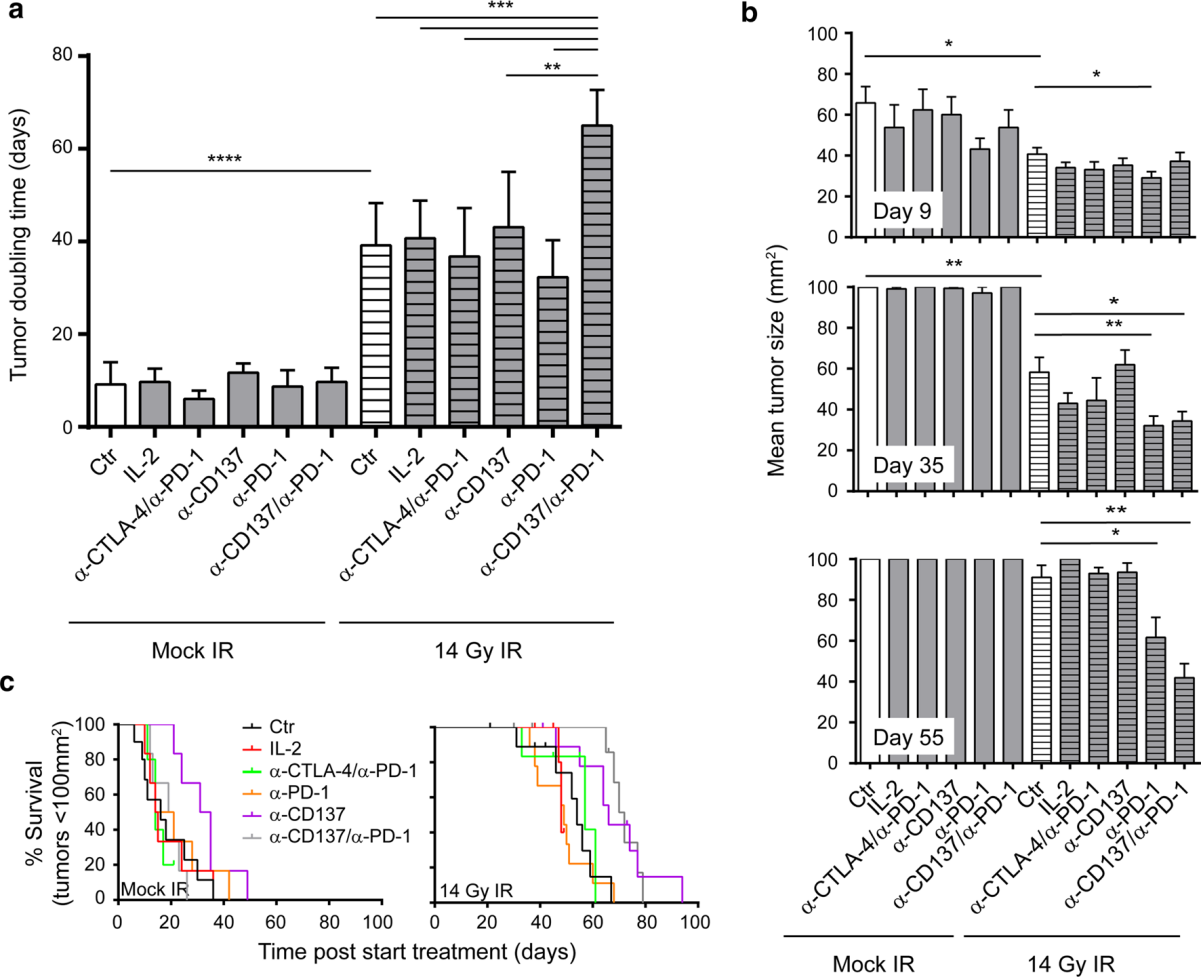
α-CD137 and α-PD-1 immunotherapy combined with SBRT reduces tumor size, delays tumor doubling time and enhances survival, when compared to mice treated with SBRT alone. **a** Quantification of tumor doubling time (from start of treatment) of all analyzable mice in Fig. 3; *bars* represent mean + SD. Statistical differences: ****p < 0.0001, ***p < 0.001, **p = 0.0011 between the indicated treatment groups. **b** Quantification of the mean tumor size of mice treated with (radio-)immunotherapy at day 9 (*top*), day 35 (*center*) and day 55 (*bottom*) post-start treatment; bars represent mean + SEM. Statistical differences *p < 0.05 between indicated treatment groups. **c** Overall survival curves for the mice analyzed in Fig. 3 for tumor growth. Survival represents the time for tumors to reach 100 mm^2^. Statistical differences between mice treated with radiotherapy (median survival 54 days) and mice treated with radiotherapy + α-CD137 and α-PD-1 mAbs (median survival 72 days) are significant (*p* = 0.005, according to log-rank Mantel–Cox test)

Treatment in all groups was well tolerated, and adverse events such as weight loss or diarrhea were not observed. None of the immunotherapeutic approaches exhibited any single-agent activity (Fig. 3). Tumor growth and mean tumor doubling times (TDTs) in these groups (IL-2: 9.7 days, α-PD-1/α-CTLA-4: 6 days, α-PD-1: 8.7 days, α-CD137: 11.7 days, α-PD-1/α-CD137: 11 days) were statistically not significant from Control-treated mice that had a mean TDT of 9.2 days (Fig. 3). SBRT-induced significant tumor growth delay (Fig. 3, 4a) and TDT increased from 9.2 days (Control) to 40.8 days (SBRT, *p* < 0.0001). IL-2 or α-PD-1/α-CTLA-4 treatment did not enhance the anti-tumor effect of SBRT (mean TDT of 40.7 (*p* = 0.71) and 36.8 days (*p* = 0.60), respectively). However, concomitant targeting of PD-1 and CD137 significantly enhanced the therapeutic effect of SBRT (mean TDT of 65.1 days compared to 40.8 days of SBRT, (*p* = 0.0006)). α-PD-1 or α-CD137 mAbs alone did not significantly enhance the anti-tumor effect of SBRT (mean TDT: SBRT + α-PD-1: 32.3 days, SBRT + α-CD137: 43.1 days vs. SBRT: 40.8 days).

Analysis of tumor sizes at different time points after initiation of therapy (day 9, day 35 and day 55) revealed an immediate effect of SBRT on tumor growth delay: At day 9, tumor sizes of all groups that received SBRT were significantly smaller compared to mock-treated mice, while immunotherapy again did not reveal any single-agent activity. At day 35, when most of tumors of non-irradiated mice had grown out, there was a statistically significant difference in tumor size between mice that received SBRT and mice that received SBRT + IL-2, SBRT + α-CTLA-4/α-CD137, SBRT + α-PD-1/α-CD137 or SBRT + α-CD137. At day 55, a time point at which most tumors treated with SBRT had started to grow out; significant differences in tumor sizes were still observed between mice treated with SBRT and mice treated with SBRT + α-PD-1/α-CD137 or SBRT + α-CD137 (Fig. 4b).

Finally, survival curves that were generated from these data also corroborated our findings: None of the immunotherapeutic approaches alone significantly extended survival compared with mock-treated mice. SBRT significantly extended survival compared to mock-treated mice (median survival SBRT: 54 days, mock treatment: 16 days, *p* < 0.0001). Concomitant targeting of PD-1 and CD137 significantly extended survival of mice that were treated with radiotherapy only (median survival SBRT: 54 days, SBRT + α-PD-1/α-CD137: 72 days, *p* = 0.0005). In this analysis, α-CD137 treatment also slightly extended survival compared to mice that were treated with radiotherapy only (median survival SBRT: 54 days, SBRT + α-CD137: 66 days, *p* = 0.02), while α-PD-1 treatment had no such effect (median survival SBRT + α-PD-1: 49 days, NS compared with SBRT only; Fig. 4c).

### CD4^+^ and CD8^+^ T cells are required for efficacy of SBRT + α-CD137 and α-PD-1 mAbs

**Fig. 5:**
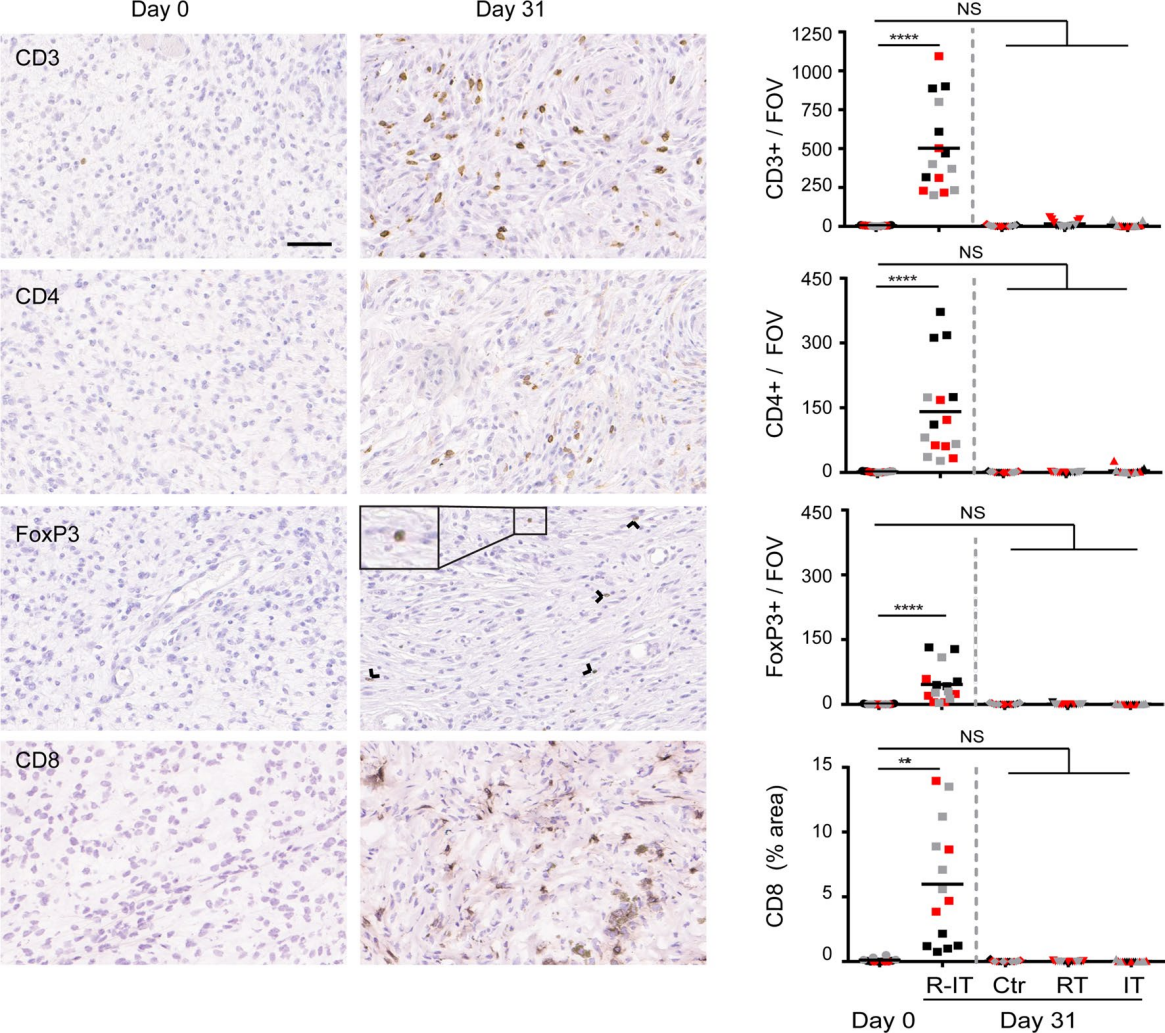
Radiotherapy + α-CD137/α-PD-1 treatment promotes presence of intratumoral T cells. Mice (three per group) bearing established melanomas were mock-irradiated or exposed to 14 Gy radiotherapy alone or in combination with α-CD137/α-PD-1 mAbs or isotype-matched Control antibody. At day 0 (D0, before treatment), or day 31 after initiation of treatment (D31), tumors were harvested, processed and stained for CD3, CD4, FoxP3 or CD8 (left panels; scale bar: 50 µM). Arrowheads point toward FoxP3-positive nuclei. For each tumor (represented with a separate color), five fields in the same section (data points) were analyzed. These data points represent number positive cells (CD3, CD4, FoxP3) or % area (CD8) per field of view (FOV), and line represents the mean. Average fold-increase CD4^+^ T cells: 141 (day 31)/1.6 (day 0) = 88. Average fold-increase CD8^+^ T cells: 6.0 % (day 31)/0.1 % (day 0) = 60. Differences between datasets were analyzed with Mann–Whitney *U* test, ****p < 0.0001; ***p = 0.0002

Next, we aimed to identify the immune cell subsets that contributed to the therapeutic effect of SBRT in combination with α-PD-1 and α-CD137 mAbs. We first analyzed tumors for immune cell infiltrates by immunohistochemistry at day 31 post-start treatment, a time point at which tumors were still in a stable phase (Fig. 3, bottom right panel). Radio-immunotherapy resulted in increased frequencies of CD4^+^ and CD8^+^ T cells in the melanomas (88-fold for CD4^+^ T cells and 60-fold for CD8^+^ T cells, Fig. 5). This effect was specific to treatment by radio-immunotherapy, since mock treatment, radiotherapy or immunotherapy alone did not increase T cell frequencies at day 31 post-start treatment. In addition, the frequency of cells expressing Granzyme B (a cytolytic effector molecule found in the granules of cytotoxic T cells and NK cells) was also significantly increased in mice treated with radio-immunotherapy compared to all other groups (Supplemental Figure 6). Finally, we observed a trend in that effector quality, on a per-T cell basis was increased in mice treated with α-CD137/α-PD-1 therapy alone or in combination with radiotherapy. This was revealed by a higher frequency of T cells expressing CD43 and producing TNF-α following PMA/ionomycin stimulation following treatment with α-CD137/α-PD-1 therapy (Supplemental Figure 7). Together, these data suggest that CD4^+^ and/or CD8^+^ T cells may contribute to controlling melanoma outgrowth by radio-immunotherapy.

**Fig. 6:**
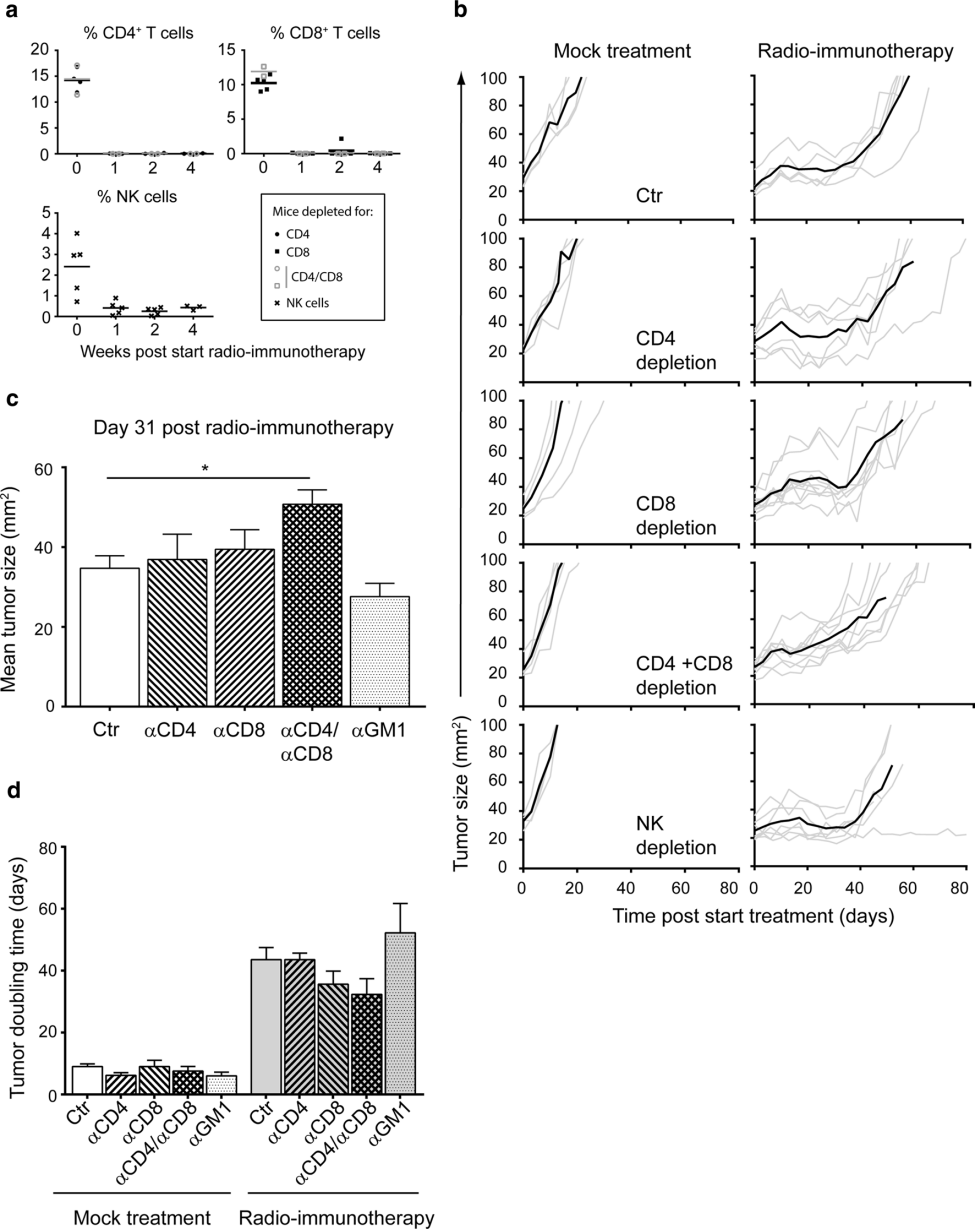
Effect of CD4, CD8 T cells and NK cell depletion to the therapeutic response of radio-immunotherapy. Mice bearing established melanomas were treated with rat IgG2a (2A3) + Rat IgG2b (LTF-2) Control Ig (Ctr), depleting antibodies to CD4 (GK1.5, rat IgG2a 250, µg twice weekly), CD8 (53.5.7, Rat IgG2b, 250 µg twice weekly) or asialoGM1 (NK cell depletion) before mock irradiation and Control Ig (Control) or radio-immunotherapy (14 Gy radiotherapy + α-CD137/α-PD-1 mAbs). **a** Validation of CD4, CD8, NK cell depletion in peripheral blood at indicated time points. Each *symbol* represents one mouse (*n* = 2–7 mice per group), and *line* represents mean. **b** Individual (*gray lines*) and mean (*black line*, terminated when >3 mice are lost from the group) tumor growth curves in indicated treatment groups are shown. **c** Quantification of the mean tumor size of mice treated with radio-immunotherapy at day 31; *bars* represent mean + SEM. Differences between mock-depleted mice and mice depleted for CD4, CD8, CD4/8, NK cells were analyzed with Mann–Whitney *U* test and considered significant for *p < 0.05. **d** Quantification of tumor doubling time (from start of treatment) of all analyzable mice in (**a**); *bars* represent mean + SEM. Differences between datasets were analyzed with Mann–Whitney *U* test and considered significant for *p < 0.05; *NS* not significant

To test this possibility, we depleted CD4^+^ and/or CD8^+^ T cells in melanoma-bearing mice just before initiating radio-immunotherapy (Fig. 6a). Combined depletion of CD4^+^ and CD8^+^ T cells reduced the therapeutic effect of radio-immunotherapy when compared to mock depletion (50.7 mm^2^ versus 34.7 mm^2^ for CD4/CD8 depletion, *p* = 0.01). In addition, despite not reaching statistical significance, the TDT of CD4^+^ and CD8^+^ T cell-depleted mice treated with radio-immunotherapy (32.3 days) was shorter compared to mock-depleted mice (43.6 days, *p* = 0.25 Fig. 6b–d). Depletion of NK cells did not alter the therapeutic effect of radio-immunotherapy (27.6 mm^2^ versus 34.7 mm^2^ for NK depletion versus mock depletion, *p* = 0.20).

Collectively, our data suggest that concomitant triggering of CD137 and blocking of PD-1 signalling within irradiated melanomas enhance the intratumoral presence of both CD4^+^ and CD8^+^ T cells, which are in part required for melanoma Control.

## Discussion

T cell checkpoint inhibitors like α-CTLA-4 and α-PD-1/PD-L1 mAbs have revolutionized treatment of melanoma [33]. However, still a large proportion of late-stage melanoma patients do not observe long-term benefit from these treatments [8–10]. In this work, we assessed how to further improve response rates by combining T cell checkpoint inhibitors with SBRT and/or T cell costimulatory molecules.

We demonstrate that combined targeting of the T cell costimulatory receptor CD137 and coinhibitory receptor PD-1 enhances the therapeutic efficacy of SBRT in a mouse model of human BRAFV600-driven melanoma. While none of our immunotherapy approaches (including α-CTLA-4/α-PD-1, α-CD137/α-PD-1, IL-2) possessed any anti-tumor efficacy themselves, only α-CD137/α-PD-1 enhanced the anti-tumor effect of SBRT. Therefore, α-CD137/α-PD-1 therapy outperformed the capacity α-CTLA-4/α-PD-1 or IL-2 treatment to synergize with SBRT in this mouse model, even though IL-2 in combination with SBRT-induced anti-tumor responses in human melanoma patients [27]. Our data therefore suggest that SBRT combined with α-CD137/α-PD-1 mAbs may be superior to the currently tested combinations of radiotherapy α-CTLA-4 or α-PD-1 mAbs. In addition, this therapeutic strategy may even benefit α-CTLA-4/α-PD-1-unresponsive patients. In addition, α-CD137/α-PD-1 therapy may synergize with other (conventional or targeted) therapeutics, such as cisplatin [34].

The enhanced anti-tumor effect of α-CD137/α-PD-1 mAbs when combined with radiotherapy was associated with accumulation (>60-fold) of intratumoral CD4^+^ and CD8^+^ T cells with an effector phenotype, which contributed to the therapeutic effect of this radio-immunotherapy approach.

However, depletion of CD4 and CD8 T cells did not completely abrogate the therapeutic effect. Taking into consideration that α-CTLA-4 or α-PD-1 mAbs did not enhance the therapeutic effect of radiotherapy, these findings indicate that CD137 triggering may also mobilize other effector mechanisms of cell types other than T cells and NK cells, including dendritic cells, monocytes, B cells, neutrophils and mast cells (reviewed in [35]). Activation of CD137 on tumor endothelial cells can augment immune cell infiltration as a result of increased adhesion to endothelial walls [36]. Furthermore, ligation of CD137 on macrophages and DCs can result in the induction of IL-8 and IL-12, respectively [35, 37]. Finally, the effect of T cell influx following our radio-immunotherapy approach may Control tumors indirectly by, for instance, reducing immunosuppressive immune cells (MDSCs) through T cell cytokines [38]. Of note, in addition to T cell infiltration in tumors following our radio-immunotherapy approach, we observed profound influx of macrophages (data not shown). We are currently functionally addressing these macrophages, as well as their importance in tumor development and therapy response.

Even though targeting CTLA-4 and PD-1 pathways with mAbs improved treatment outcomes for late-stage melanoma patients [33], we did not observe any therapeutic effect of these antibodies in our mouse model. Responses to T cell checkpoint blockade have recently been correlated to the mutational load of the tumor and its associated immunogenicity [39]. The mouse model we used lacks this mutational load as it is not induced by UV irradiation as human melanoma, but by the deliberate introduction of two genetic alterations, namely loss of *Pten* and gain of mutant *Braf*. As a result, the tumors induced in this model are probably less immunogenic than tumors arising in melanoma patients, likely explaining the absence of responses upon treatment with CTLA-4, PD-1 mAbs, IL-2 alone (Fig. 3) or in combination with targeted agents [26]. Therefore, the enhanced effect of targeting CD137 and PD-1 in combination with radiotherapy in this—poorly immunogenic—model likely underestimates the potential of this therapy in melanoma patients.

In conclusion, we observed significant improved anti-tumor efficacy by combining radiotherapy with α-CD137 and α-PD-1 mAbs. We observed this in a poorly immunogenic mouse model of human melanoma, which did not respond to α-PD-1 mAbs alone or in combination with α-CTLA-4 mAbs. This observation indicates that the combination of α-PD-1 and α-CD137 might be more powerful than currently used α-PD-1 alone or in combination with α-CTLA-4 blockade in human melanoma. In addition, our study suggests that radiotherapy in combination with α-PD-1 and α-CD137 mAbs may be superior to the currently tested combinations of radiotherapy with α-CTLA-4 or α-PD-1 mAbs. In addition, this therapeutic strategy may even benefit α-CTLA-4/α-PD-1-unresponsive patients, which should both be tested clinically.

### Electronic supplementary material

Below is the link to the electronic supplementary material.

Supplementary material 1 (PDF 2387 kb)

## Abbreviations

AEC: 3-Amino-9-ethylcarbazole
APC: Allophycocyanin
CT: Computed tomography
DAB: Diaminobenzidine
FOV: Field of view
Gy: Gray
kVp: Peak kilo voltage
OCT: Optimal cutting temperature compound
SBRT: Stereotactic body radiation therapy
SEM: Standard error of the mean
TDT: Tumor doubling time
