# Supplementary material for: Concomitant targeting of programmed death-1 (PD-1) and CD137 improves the efficacy of radiotherapy in a mouse model of human BRAFV600-mutant melanoma
Source: Cancer Immunol Immunother. 2016 May 9;65(6):753–63. doi: 10.1007/s00262-016-1843-4 (PMC4880641; doi:10.1007/s00262-016-1843-4)
Supplement: Supplementary file 1 — Supplementary material 1 (PDF 2387 kb) [file 262_2016_1843_MOESM1_ESM.pdf]

Kroon et al., Supplemental Figure 1

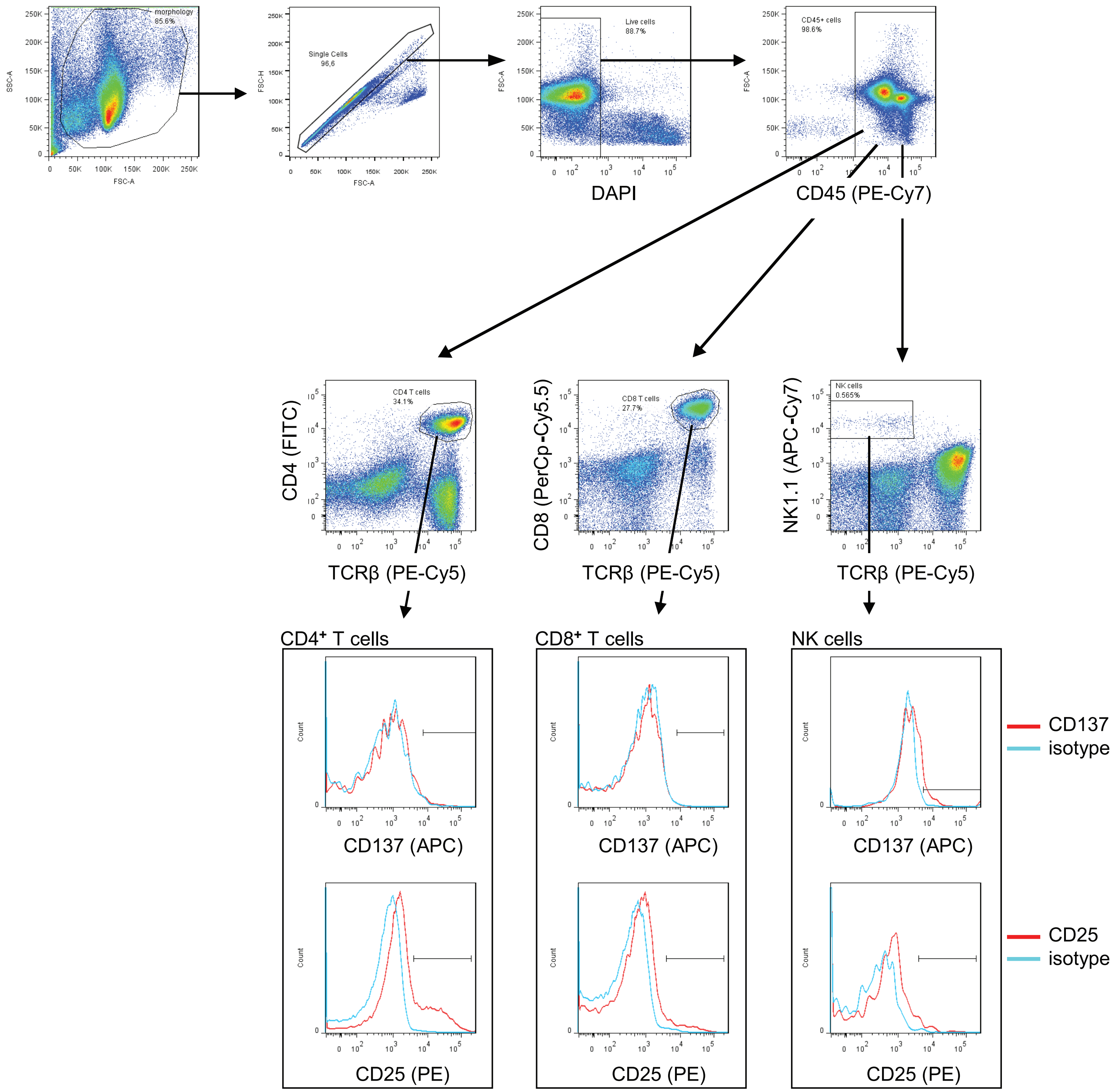

Supplemental Figure 1. Gating strategy for lymph node samples. Example of gating strategy for the detection of CD25 and CD137 cell surface markers on CD4+ T cells, CD8+ T cells and NK cells in lymph node samples (see also Materials and Methods)

Kroon et al., Supplemental Figure 2

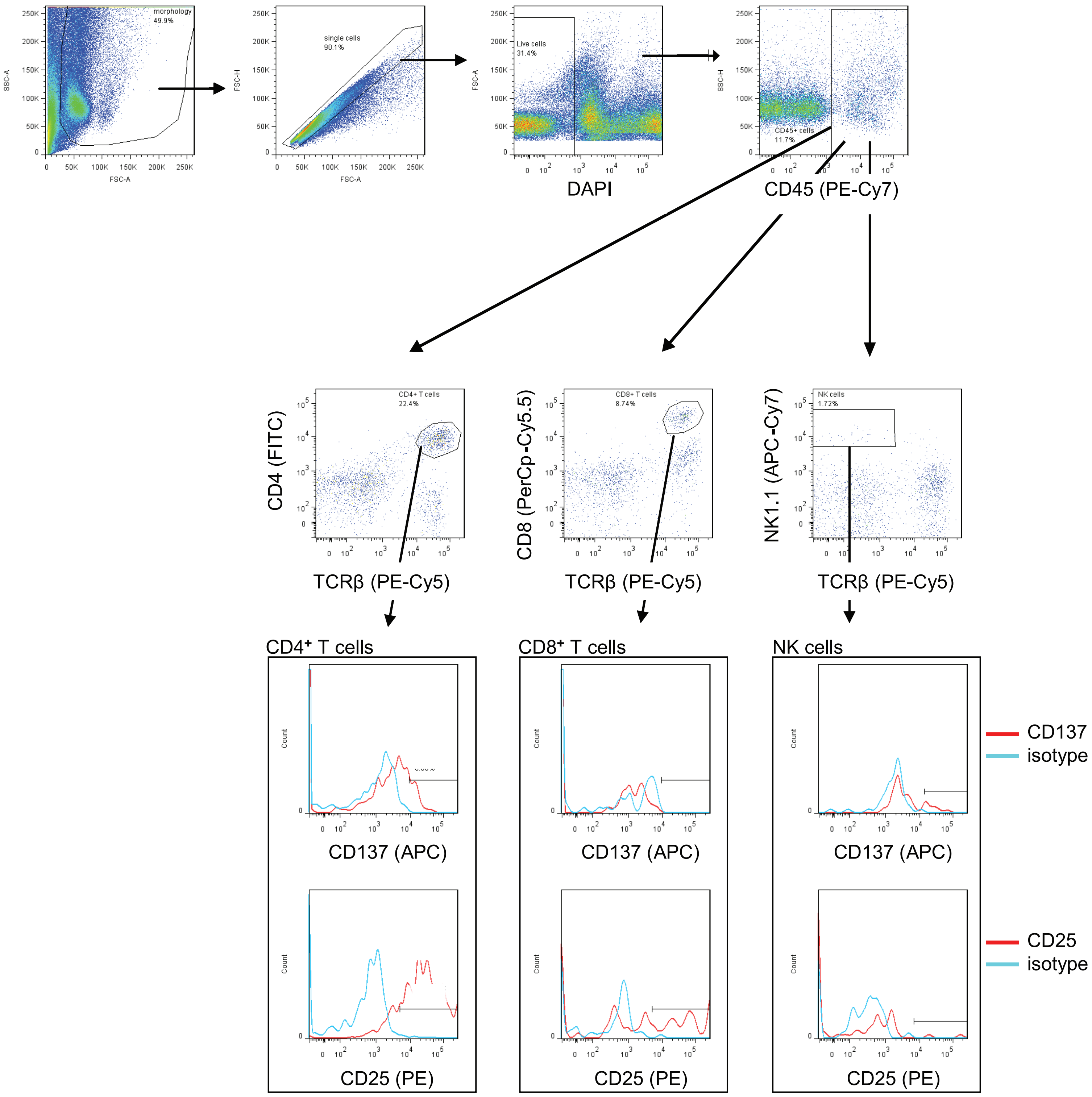

Supplemental Figure 2. Gating strategy for tumor samples. Eample of gating strategy for the detection of CD25 and CD137 cell surface markers on CD4+ T cells, CD8+ T cells and NK cells in tumor samples (see also Materials and Methods)

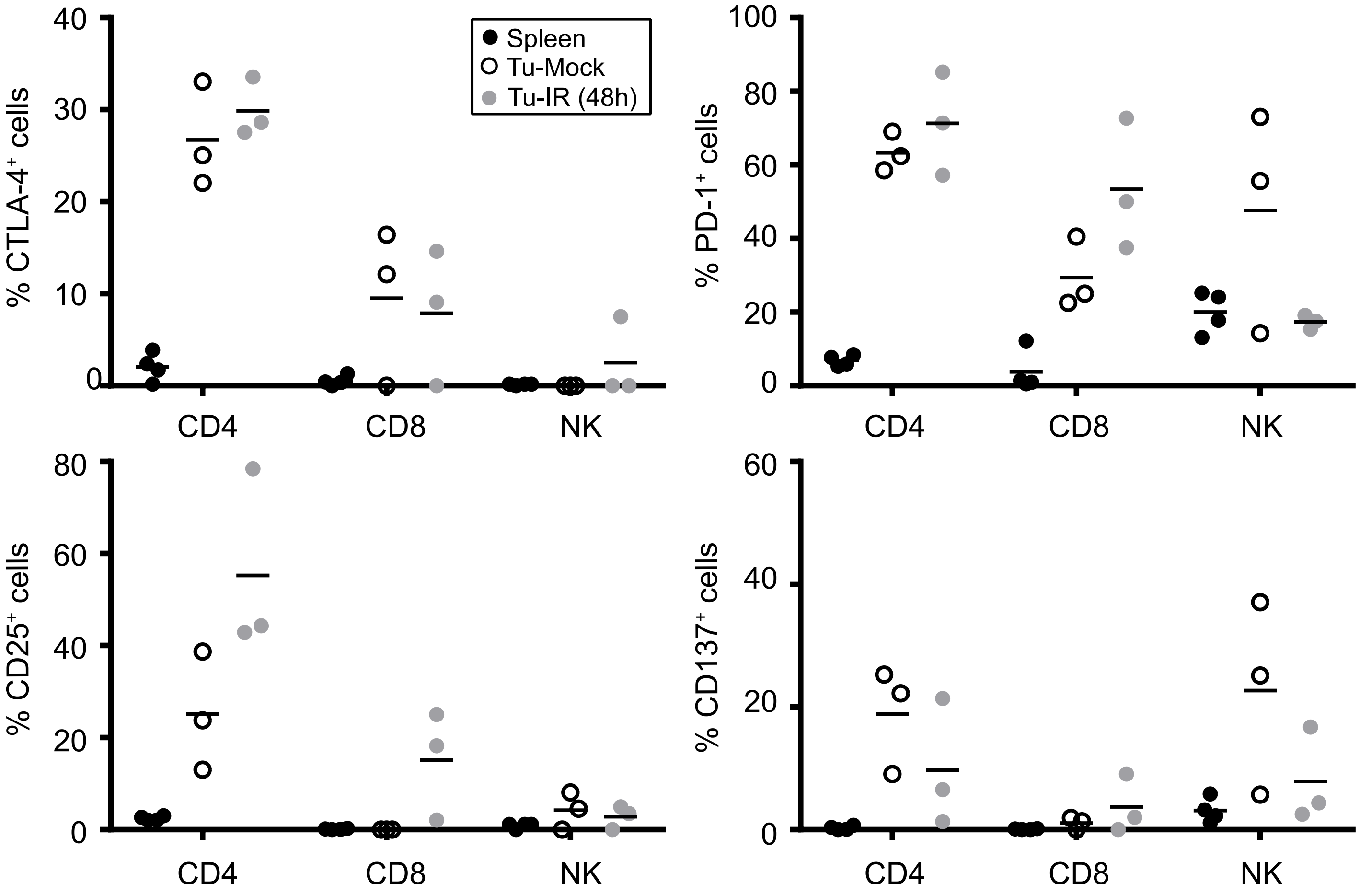

Supplemental Figure 3. Radiotherapy (48 hours) does not alter expression of CTLA-4, PD-1, CD25 and CD137 on TILs. Mice (3 per group) bearing established melanomas were mock-irradiated or treated with 14 Gy radiotherapy. After 48 hours, tumors were harvested, processed and stained for CD45 (eVolve-610), TCRb (Pe-Cy™5), CD4 (FITC), CD8 (eFluor® 450), NK1.1 (eVolve655), CTLA-4 (PE-eFluor® 610), PD-1 (PE), CD25 (AlexaFluor® 700), CD137 (APC), or the corresponding isotype controls (which were subtracted). Fixable IR-dye was used as viability dye. Data represent the net frequency of cells expressing CTLA-4, PD-1, CD25 and CD137 per mouse.

a

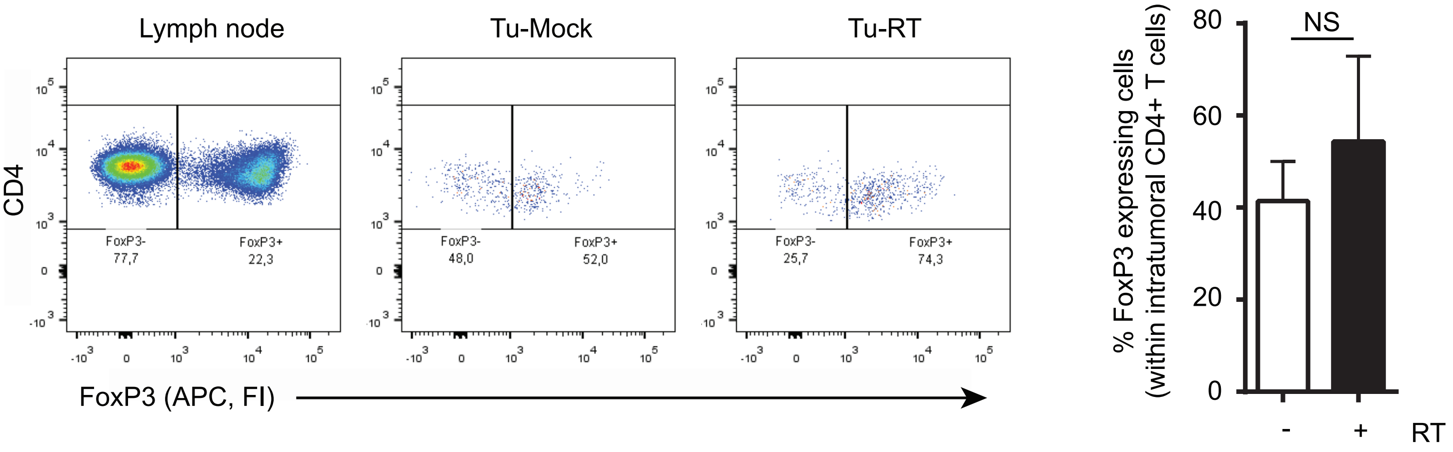

b

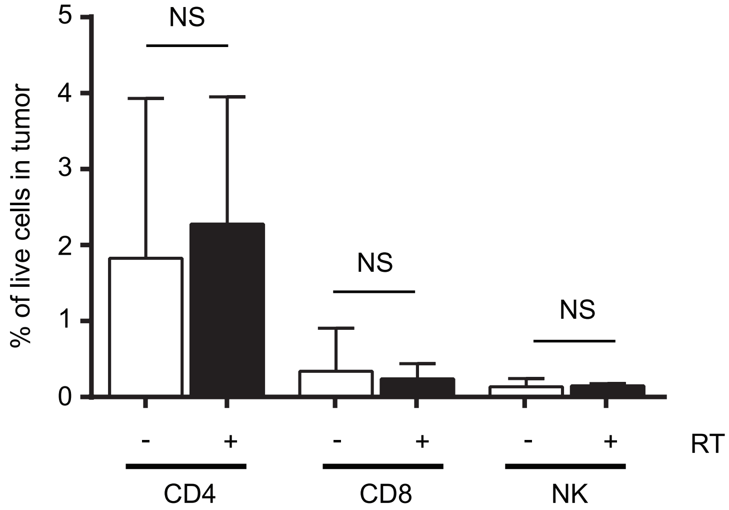

Supplemental Figure 4. Radiotherapy does not alter the frequency of T cells and NK cells in melanoma. a. Flow cytometric analysis of FoxP3 expression within CD4+ T cells derived from lymph node (LN), mock-irradiated tumor (Tu-mock) or 14 Gy irradiated tumor (Tu-RT). Quantification of 3-4 tumors is presented in the histogram. All data is presented as mean +SD. Differences between data-sets were analyzed with Mann-Whitney U-test and considered significant for $p < 0.05$; NS: not significant ($p = 0.6286$). **b.** Frequency of CD4+ T cells, CD8+ T cells and NK cells as percentage of live cells (Gating: single cells, DAPI-) in mock-irradiated tumors (- RT) and 14 Gy irradiated tumors (+ RT). Data is derived from the same samples presented in Figures 1-2.

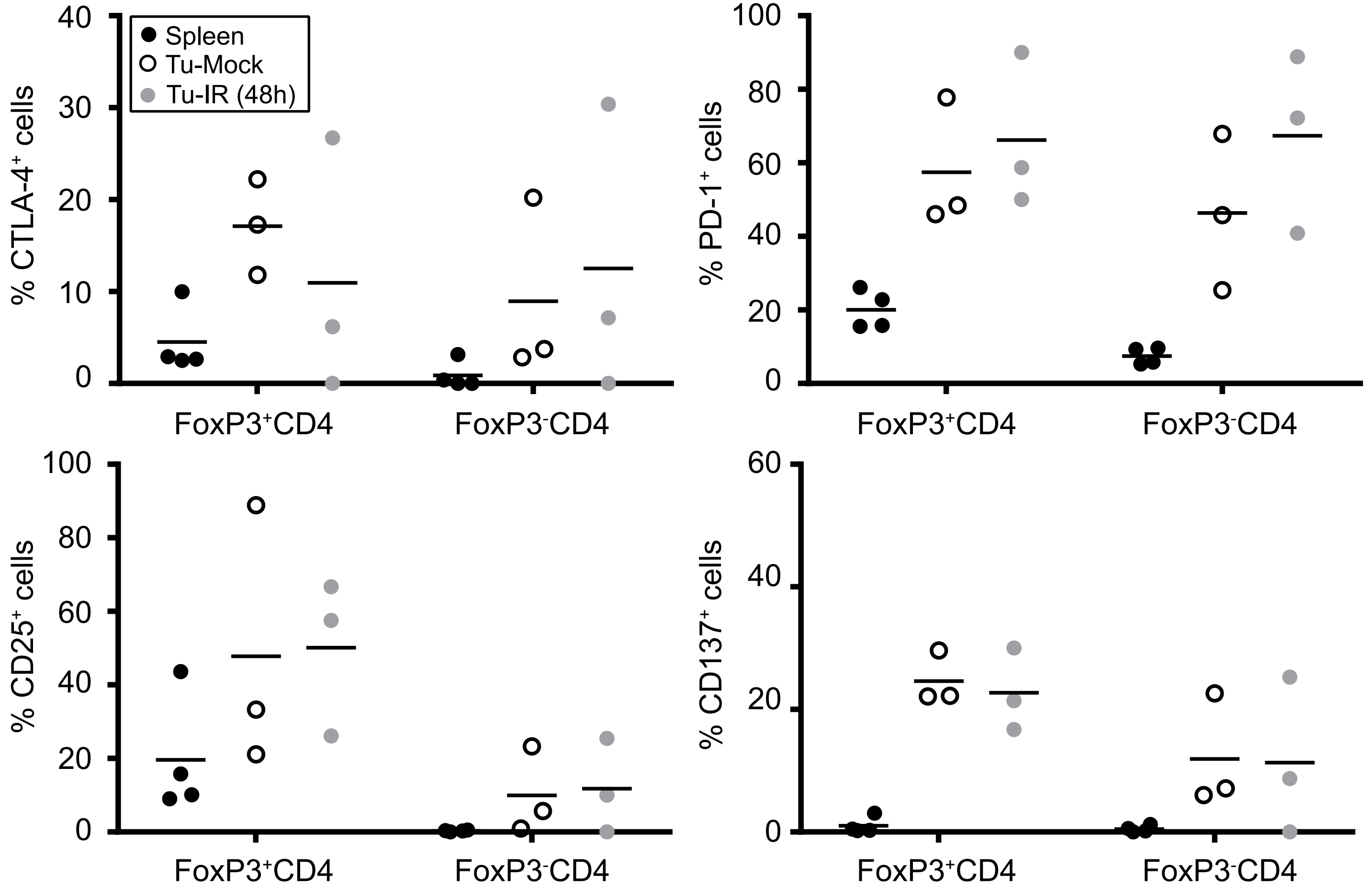

Supplemental Figure 5. Expression of CTLA-4, PD-1, CD25 and CD137 on Regulatory versus non-regulatory CD4+ T cells. Mice (3 per group) bearing stablished melanomas were mock-irradiated or treated with 14 Gy radiotherapy. After 48 hours, tumors were harvested, processed and stained for the same molecules as in Supplementary Figure 4, then fixed, permeabilized and additionally stained with an antibody to FoxP3 (PE-Cy7) to discriminate regulatory versus 'non-regulatory' CD4+ T cells. Expression of CTLA-4, PD-1, CD25 and CD137 was determined as described for Supplementary Figure 4. Data represent the net frequency of cells expressing CTLA-4, PD-1, CD25 and CD137 per mouse.

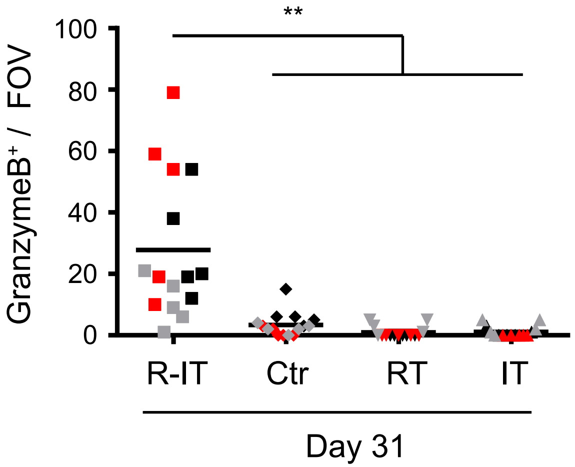

Supplemental Figure 6. Radio-immunotherapy increases frequency intratumoral cells expressing Granzyme B. Mice (3 per group) bearing established melanomas were mock-irradiated or treated with 14 Gy radiotherapy alone or in combination with α-CD137/α-PD-1 mAbs or isotype-matched control antibody. At day 31 after initiation of treatment tumors were harvested, processed and stained foror Granzyme B. Data points represent number positive cells per field of view (FOV), and line represents the mean. Each tumor is represented with a separate colour. Differences between datasets were analysed with Mann-Whitney U-test, **$p < 0.01$.

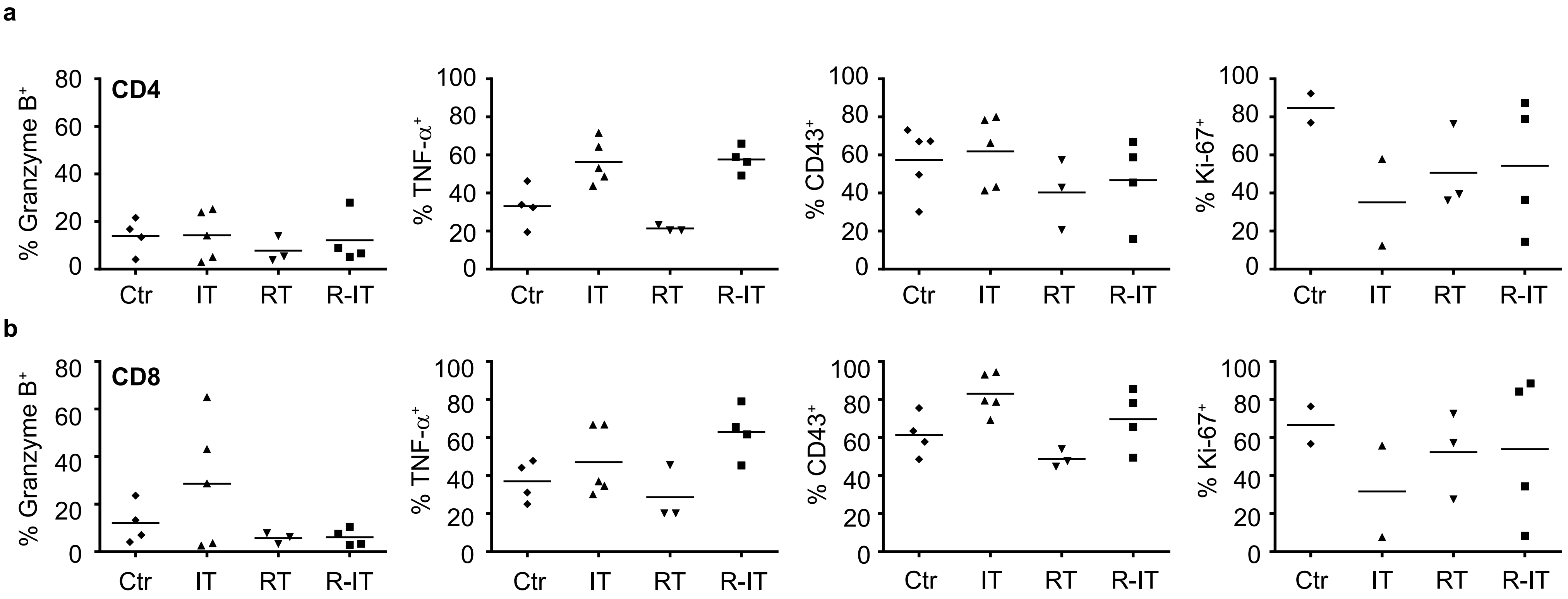

Supplemental Figure 7. T cell quality in mice treated with radiotherapy + α-CD137/α-PD-1 treatment. Mice (3-5 per group) bearing established melanomas were mock-irradiated or treated with 14 Gy radiotherapy alone or in combination with α-CD137/α-PD-1 mAbs or isotype-matched control antibody. On day 20 after initiation of treatment (a time-point before mice required sacrification due to large tumor size), tumors were harvested, processed, stimulated with Phorbol 12-myristate 13-acetate (PMA, 50 nM) and Ionomycin (1 μM) for 3,5 hours and stained for CD45, CD3, CD4, CD8, Granzyme B, TNF-α, Ki-67 and CD43. Datapoints represent the frequency of positive CD4 (**a**), and CD8 (**b**) cells per tumor, and the line represents the mean.
